# Trop2-targeted therapy in breast cancer

**DOI:** 10.1186/s40364-024-00633-6

**Published:** 2024-08-13

**Authors:** Yixuan Hu, Yinxing Zhu, Dan Qi, Cuiju Tang, Wenwen Zhang

**Affiliations:** 1 Department of Oncology, Nanjing First Hospital, Nanjing Medical University, Nanjing, 210006 China; 2 Department of Radiation Oncology, The Affiliated Huaian No. 1 People’s Hospital of Nanjing Medical University, Huai’an, China; 3 Women’s Hospital of Nanjing Medical University, Nanjing Women and Children’s Healthcare Hospital, Nanjing, China

**Keywords:** Trop2, Breast cancer, TNBC, SG, Antibody-drug conjugate, Targeted therapy

## Abstract

Human trophoblastic cell surface antigen 2 (Trop2) is a glycoprotein, a cellular marker of trophoblastic and stem cells, and a calcium signaling transducer involved in several signaling pathways, leading to the proliferation, invasion, and metastasis of tumors. It is expressed at a low level in normal epithelial cells, but at a high level in many tumors, making it an ideal target for cancer therapy. According to previous literature, Trop2 is broadly expressed in all breast cancer subtypes, especially in triple negative breast cancer (TNBC). Several clinical trials have demonstrated the effectiveness of Trop2-targeted therapy in breast cancer. Sacituzumab govitecan (SG) is a Trop2-targeted antibody-drug conjugate (ADC) that has been approved for the treatment of metastatic TNBC and hormone receptor-positive (HR+) and human epidermal growth factor receptor 2-negative (HER2-) breast cancer. This article reviews the structure and function of Trop2, several major Trop2-targeted ADCs, other appealing novel Trop2-targeted agents and relevant clinical trials to provide a landscape of how Trop2-targeted treatments will develop in the future.

## Introduction

The most common diagnosed cancer among women is breast cancer [1]. According to the molecular analysis of gene expression profiling, it can be divided into several molecular subtypes: luminal A (estrogen receptor-positive (ER+) and/or progesterone receptor (PR+), HER2-, Ki67 + < 20%), luminal B (ER + and/or PR+, HER2-, Ki67 + ≥ 20%), HER2 over-expression (ER-, PR-, HER2 over-expression), basal-like, and other special subtypes [2]. Basal-like breast cancer typically presents as ER-, PR-, and HER2- (triple-negative breast cancer, TNBC), but not all of them are triple-negative. Some basal-like breast cancers may have low-level expression of ER or PR. About 50 − 75% of TNBC exhibit a basal phenotype, and 80% of basal-like tumors fall under the category of TNBC [3, 4]. TNBC accounts for approximately 20% of newly diagnosed breast cancers, characterized by high aggressiveness with greater metastasis potential and unfavorable clinical prognosis [5]. Due to the lack of clear molecular targets, TNBC is not sensitive to endocrine therapy or molecular targeted therapy, and chemotherapy is still the first choice of treatment. Therefore, the new treatment and targets of TNBC is considered to be a very challenging topic. In 1981, a highly expressed protein on the surface of trophoblast cells was identified as trophoblastic cell surface antigen 2 (Trop2) [6]. It is expressed in most subtypes of breast cancer, especially in TNBC, but low or even absent in normal tissues [7]. Sacituzumab govitecan (SG) is a typical Trop2-targeted antibody-drug conjugate (ADC), and the Food and Drug Administration (FDA) has approved SG for the treatment of TNBC in April 2020 at an accelerated rate. More recently, it was granted permission for the treatment of HR+/HER2- breast cancer [8]. Given the success of SG, other ADCs targeting Trop2 are being explored. In this article, we will clarify the mechanism of action of Trop2 and its capacity in the treatment of breast cancer.

## The role of Trop2 in breast cancer

### Structure and mechanism of Trop2

**Fig. 1:**
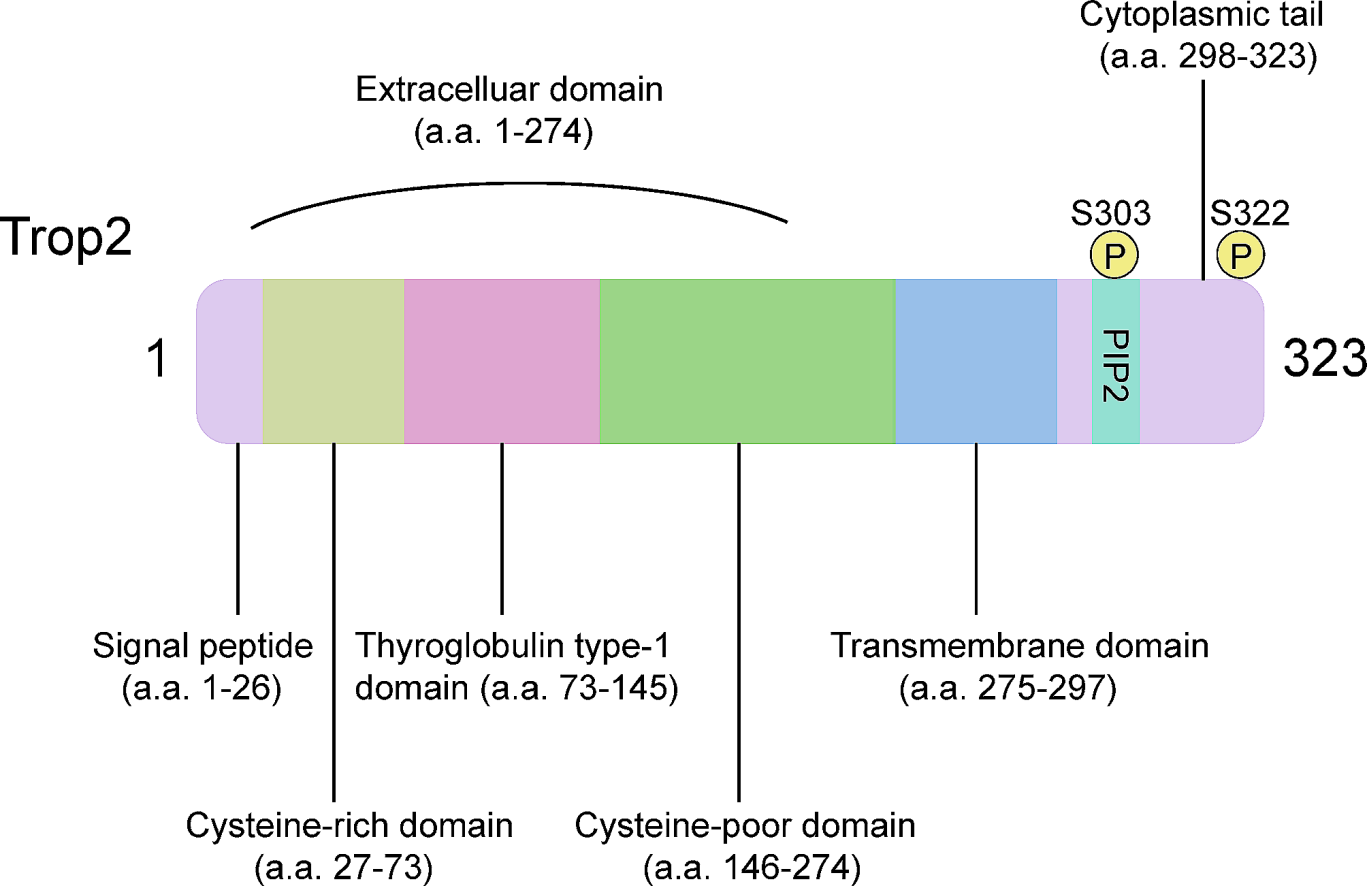
Structural characteristics of Trop2. The extracellular domain of Trop2 includes a cysteine-rich domain, a cysteine-poor domain and a thyroglobulin type-1 domain. The cytoplasmic tail includes a PIP2 binding sequence and two phosphorylation sites (S303 and S322)

Trop2 is a type I cell surface glycoprotein, also known as tumor-associated calcium signal transducer 2 (TACSTD2), which is a cellular marker for trophoblast and stem cells and is critical for embryonic development. Trop2 is expressed at low levels on the surface of normal epithelial cells and at high levels in many epithelial tumors, such as colon, pancreatic and breast cancer, making it an ideal target for cancer therapy [9]. Trop2 gene encodes a 35–46 kDa protein, containing an extracellular domain (ECD), a single transmembrane domain and a short cytoplasmic tail [10]. Trop2-ECD includes 1 unique Cysteine-Rich Domain (CRD), 1 Tyrosine Cluster Domain (TY), and 1 Cysteine-deficient Domain (CPD), enabling the formation of a stable dimer [11] (Fig. 1). This structure regulates signal transduction and affects the progression of the cell cycle [12].

**Fig. 2:**
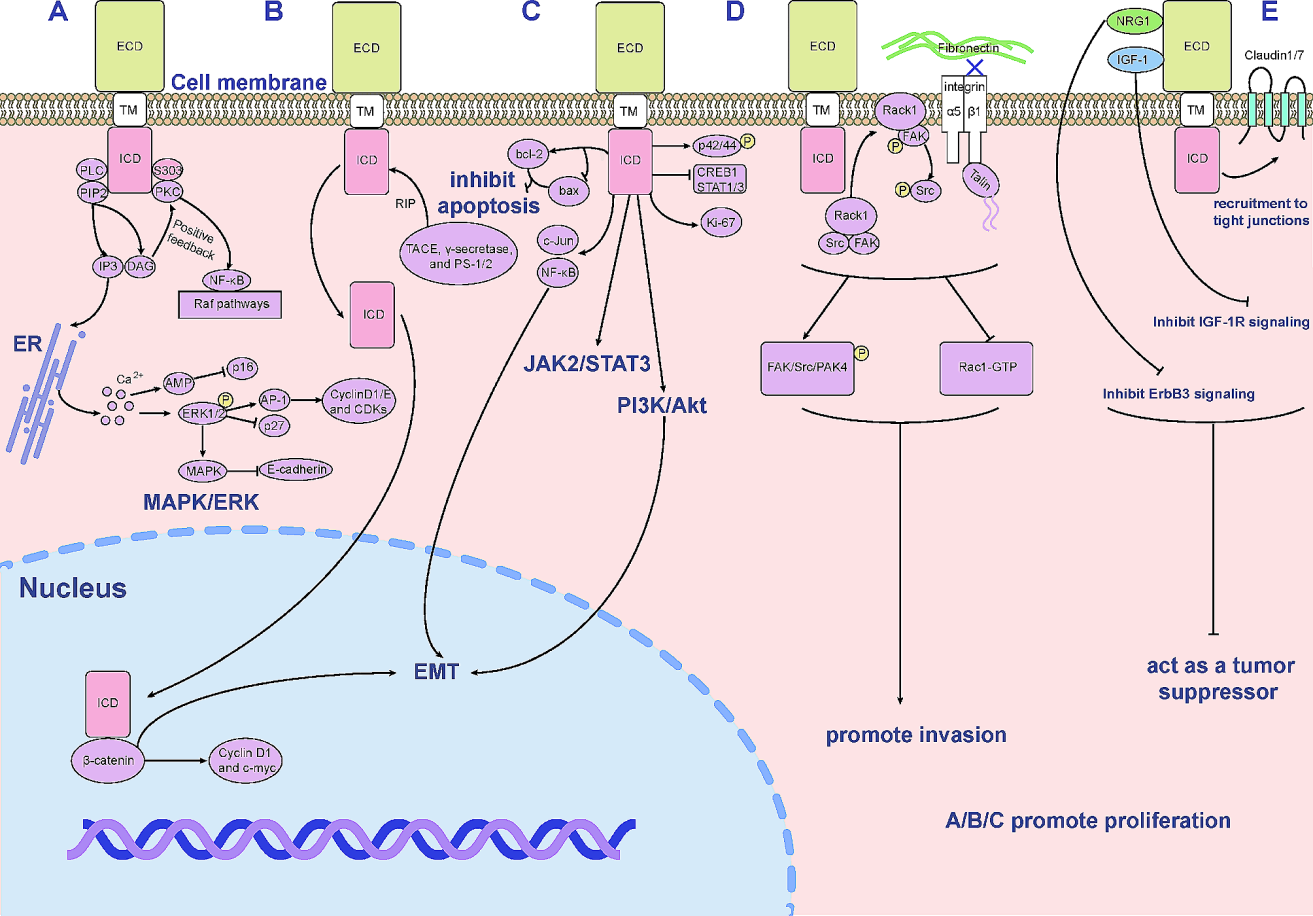
Trop2-mediated signaling pathways. (**A**) Protein kinase C (PKC) can phosphorylate serine residue (S303) of the cytoplasmic tail of Trop2. Diacylglycerol (DAG) and inositol triphosphate (IP3) are created by the hydrolysis of phosphatidylinositol 4,5-bisphosphate (PIP2). IP3 can bind to specific endoplasmic reticulum receptors, release Ca2 + deposited there, increase intracellular calcium concentrations, and thus activate MAPK signaling pathways and promote cell proliferation. Ca2 + inhibits p16 expression through the AMP-activated protein kinase pathway. Increased phosphorylation of ERK1/2 enhances AP-1 and inhibits p27. The enhancement of AP-1 activity increased the expression of cyclin D1/E and CDKs. Trop2 also inhibits the expression of E-cadherin by stimulating the MAPK signaling pathway. In addition, DAG activates more PKC through a positive feedback mechanism, which increases Trop2 phosphorylation, thereby stimulating the Raf pathway and NF-κB. (**B**) Trop2 is hydrolyzed to ICD by TACE, γ-secretase, and PS-1/2. After the shed of Trop2-ECD, the co-localization of Trop2-ICD and β-catenin in the nucleus can induce the up-regulation of cyclin D1 and proto-oncogene c-myc downstream, thus promoting cell proliferation. (**C**) Trop2 overexpression leads to increased phosphorylation of p42 and p44 and increased Ki-67 levels. Trop2 inhibits apoptosis by increasing the expression of bcl-2 and decreasing the expression of bax. Trop2 also upregulates transcription factors (NF-κB and c-Jun) and downregulates transcription factors (CREB1, STAT1 and STAT3). Trop2 is involved in other signaling pathways, such as PI3K/Akt and JAK2/STAT3 signaling pathways. (**D**) Trop2 promotes the aggregation of RACK1 on the cell membrane, interacts with integrin β1 and talin, and promotes the localization of integrin α5β1/talin complex to the cell leading edge, away from focal adhesions. It also increases phosphorylation of kinases FAK, Src, and PAK4, and decreases Rac1-GTP levels, thus promoting invasion. (**E**) Trop2 can directly bind to the transmembrane proteins claudin1 and claudin7. Phosphorylation of Trop2 serine 322 leads to the relocalization of claudins, which leads to tumor cell migration. By interacting with NRG1 and IGF-1, Trop2 inhibits ErbB3 and IGF-1R signaling pathways, respectively, and thus exerts a tumor inhibitory effect

According to previous literature, we have known Trop2 has been implicated in numerous intracellular signaling pathways [13]. Initially, Trop2 acts as a calcium signal transducer to increase intracellular Ca2^+^ concentration, activating the ERK1/2-MAPK axis and promoting cell proliferation by regulating the transcription of cell cycle genes downstream of the ERK pathway [14] (Fig. 2A). Moreover, Trop2 can promote cell proliferation by regulating intramembrane proteolysis (RIP), especially in prostate cancer [15]. When Trop2 intracellular domain (ICD) and β-catenin co-localize in the nucleus after Trop2-ECD is shed, this leads to the upregulation of downstream cyclin D1 and the proto-oncogene c-myc, thereby causing cancer progression [16]. It was reported that this cleavage event of Trop2 occurs only in tumors, but not in benign normal tissues (Fig. 2B). Beyond these, Trop2 was also found to promote tumor cell invasion through different mechanisms. By encouraging RACK1 and integrin β1 interaction, ectopic expression of Trop2 in prostate cancer cell lines reduced the adhesion to fibronectin. Also, Trop2 induces anti-adhesive and promotes motility states by increasing phosphorylation of the kinases FAK, Src, and PAK4, co-localizing with pAkt, and decreasing Rac1-GTP levels (Fig. 2C) [17, 18]. In addition, the upregulation of MMP2 via ERK and JNK pathways can amplify the pro-invasion effect of Trop2 in thyroid cancer [19]. In colorectal cancer, the ability of tumor cells to migrate depends on the phosphorylation of Trop2 serine 322, leading to the relocation of claudin7 [20]. Claudin1 and 7 are two transmembrane proteins that form tight junctions on the epithelial surface. Trop2 can bind directly to these transmembrane proteins, either protecting them from degradation by the ubiquitin-proteasome system or transporting them to the cell membrane. (Fig. 2D) [21]. The efficacy of small molecule inhibitors AG1024 targeting IGF-1R and Crizotinib targeting ALK in HeLa cells with and without Trop-2 overexpression was evaluated. Results from cell viability and migration assays revealed that the oncogenicity of vector-transfected cells was significantly more suppressed by the inhibition of IGF-1R or ALK compared to Trop2-overexpressed cells [22].

As aforesaid, Trop2 is widely recognized as an oncogene that promotes tumor growth in a variety of cancers and is positively correlated with metastasis and poor prognosis [23]. However, Trop2 can also act as a tumor suppressor under specific circumstances. In lung adenocarcinoma cell line H1299 [24], Trop2 over-expression decreases ERK and AKT phosphorylation, Trop2 binds to IGF-1, inhibiting its ability to activate IGF-1R and reducing the activation of AKT and ERK by IGF-1R signaling pathway [25]. (Fig. 2D). In addition, decreased Trop2 expression was found in liver fluke-associated cholangiocarcinoma [26] and hepatocellular carcinoma [27]. When compared to the controlled cells, Trop2 knockdown modestly increases proliferation in the MCF7 breast cancer cell line [28]. Association of tumor Trop2 expression with prognosis varies among lung cancer subtypes, and the presence of Trop2 on the cellular membrane in breast cancer indicates unfavorable overall survival (OS), while the presence of Trop2 inside the cell has a positive effect on prognosis. Besides, Trop2 has been expressed at different levels in the normal tissues, such as the epithelial barrier/lining of the stratum basale epidermis, breast, cervix, cornea, the epithelial secretory tissue of the endocrine and exocrine glands, esophagus, heart, prostate, skin, uterus and so on [29]. These findings imply that the regulation of proliferation by Trop2 is a complicated, cell-type and organ-specific phenomenon. The exact role of Trop2 may depend on the broader genetic background of a particular cancer cell.

### Trop2 as a therapeutic target in breast cancer

Studies have evaluated Trop2 protein levels in breast cancer tumor tissues and discovered that high Trop2 expression has been found in samples with 93% TNBC (*n* = 28), 74% HER2+ (*n* = 35), and 50% ER+ (*n* = 22) [30]. Furthermore, Vidula et al. investigated I-SPY1, METABRIC, and TCGA databases and found that Trop2 was widely expressed in all breast cancer subtypes, especially luminal A and TNBC [31]. These results lend credence to the potential of Trop2 as a therapeutic target for multiple breast cancer subtypes.

Aslan et al. implanted HCC1806 TNBC cells with downregulated or deleted Trop2 gene into female NSG mice, finding that downregulation or deletion of Trop2 led to a significant delay in tumor growth and a reduction in tumor weight [30]. In addition, the ability of cells to form colonies was greatly enhanced after over-expression of Trop2 in MCF7, an ER + breast cancer cell line with low levels of endogenous Trop2. Further studies found that Trop2 may mediate metabolic reprogramming and induction of oncogenic proteins in TNBC [30]. Five metabolic genes (LDHA, SHMT2, GPI, ADK, and TALDO1) had increased mRNA levels in TNBC patients compared to ER + or HER2 + patients, according to their analysis of the METABRIC dataset. These genes were downregulated in Trop2 depleted tumors. By studying the correlation between the metabolic characteristics of these five genes and clinical outcomes, it was found that increased mRNA of these five genes predicted poor prognosis and overall survival (OS) of early breast cancer [30]. Similarly, Ambrogi et al. analyzed the association between Trop-2 expression and outcomes of 702 breast cancer patients, and found that patients with higher membranous expression of Trop-2 had a worse prognosis (overall survival and disease relapse) [32].

To investigate whether Trop2 can be used as a target site for the treatment of TNBC, Lin et al. isolated Trop2 Fab that could specifically recognize and bind Trop2-ECD using a human Fab phage library. They found that Trop2 Fab efficiently and dose-dependently suppressed proliferation and induced apoptosis in the TNBC cell line. The antitumor effect of the Trop2 Fab was also confirmed in a breast cancer xenograft model [33].

## Preclinical research of Anti-Trop2 therapy in breast Cancer

Several Trop2-targeted drugs are currently under development, including antibodies, ADCs, and combination therapies, with ADCs at the forefront of cancer therapy and precision medicine. The FDA has approved SG for the treatment of TNBC and HR+/HER2-breast cancer patients. There are several Trop2-targeted ADCs undergoing clinical trials right now, such as SG, datopotamab deruxtecan, SKB-264, JS-108, ESG-401, and FDA018. Still in the preclinical or discovery stages are other emerging medications. Here, we summarize the current state of knowledge concerning the Trop2-targeted therapy in breast cancer.

### Trop2-targeted ADCs

ADCs consist of monoclonal antibodies that directly bind to membrane antigens, along with cytotoxic agents (payloads) and linkers. The payload itself is too toxic to be used directly in clinical therapy. By precisely targeting tumor cells with monoclonal antibodies, ADCs offer a way to reduce off-target toxicity in patients by limiting normal tissue exposure to the payload, thus expanding the therapeutic window compared to conventional chemotherapy [34].

Antibodies recognize membrane antigens, and then after antigens internalization, deliver the payload to the tumor cell. Following the degradation of antibodies and the hydrolysis of linkers by lysosomes, highly toxic payloads are released into the cytoplasm, causing tumor cells to undergo apoptosis [35–37]. Additionally, payloads can be delivered to nearby cancer cells, causing the bystander effect. Even in the absence of target antigens, the bystander effect still has an impact on nearby cancer cells [38, 39]. Besides, some ADCs do not support the bystander effect because they have non-cleavable linkers [40]. Intriguingly, the adverse effects of ADCs mainly come from these components [41]. The toxicity generated by antibody varies significantly depending on the target, indication, etc., while the toxicity of payloads manifests in the damage to rapidly proliferating healthy cells. Unstable linkers lead to early release of payloads into circulation, resulting in higher peak concentrations of cytotoxicity and increased chemotherapy-related toxicity. Released payloads can also enter adjacent non-malignant cells through passive diffusion or transporter-mediated uptake, potentially causing off-target cell toxicity. Tumor heterogeneity or failure in internalization and trafficking pathways may lead to drug resistance [42]. The underlying mechanism is not yet fully understood, but altering drug structures or using different linkers to connect bispecific payloads or combination therapy can help overcome resistance and maximize the therapeutic advantages of ADCs. These challenges continue to drive the optimization of ADC design [43]. For example, antibodies of ADCs require weak immunogenicity, high targeting, high binding affinity, long half-life, and good cyclic stability [44, 45]. The effectiveness and side effects of ADCs are determined by the payloads’ mechanisms of action, so it is important to ensure that the molecular weight is low to minimize the possibility of immunogenic reactions. Also, payloads should be soluble when exposed to physiological circumstances and have a functional group that binds to antibodies via linkers [46]. Linkers affect the drug-antibody ratio (DAR) value (the amount of payload attached to the antibody), therapeutic index (TI), and pharmacokinetics/pharmacodynamics of ADCs [44]. Thus, linkers need to remain stable in the blood and, once transported to the lysosome, should quickly break down and release the payload [34, 47].

#### Sacituzumab govitecan (Trodelvy, IMMU-132)

SG is composed of hRS7 IgG1κ, coupled to SN-38 by a cleavable CL2A linker. SN-38 can cause DNA double-strand breaks, which is an active metabolite of irinotecan and a topoisomerase I inhibitor [48, 49]. SG is characterized by a high DAR, which allows high concentrations of SN-38 to be delivered. After connecting to Trop2, hRS7 (in conjugated or free form) is internalized and transported within the cell to the lysosome and SN-38 is released during antibody degradation [50]. In addition, an acidic environment hydrolyzes pH-dependent linkers surrounding the tumor, and the bystander effect kills neighboring tumor cells through the release of SN-38 [49, 51].

A clinical pharmacology study of SG recommended the dose and regimen for SG is 10 mg/kg on days 1 and 8 of 21-day cycles until the disease progresses or there is intolerable toxicity. The elimination half-life of SG and hRS7 IgG1κ are 11 ∼ 14 h and 103 ∼ 114 h, respectively. It indicates that the clearance rate of SG is faster than hRS7 IgG1κ. Moreover, the concentration of free SN-38 accounts for only 2.5% of the total SN-38, indicating that the majority (> 95%) of SN-38 in the serum is bound to antibodies, demonstrating good binding affinity and higher safety [52].

SG was evaluated in various human epithelial cancer xenograft models, demonstrating positive results compared to SN-38, irinotecan, or non-targeting ADCs in almost all of the models [50, 53, 54]. IMMU-132 was tested in mice bearing a human gastric carcinoma xenograft. Out of seven mice in the IMMU-132 group, six showed partial responses that lasted for over 18 days. The mean time to progression (TTP) was greatly extended compared to the non-targeting ADC group (41.7 ± 4.2 vs. 4.1 ± 2.0 days) [54]. Nevertheless, the SK-MES-1 lung cancer cell line, which was derived from squamous cell carcinoma, was the exception. In this cell line, the efficacy of SG was not distinct from that of irinotecan [50]. It worth of further clinic confirmation. In order to examine how antigen expression affects SG efficacy in vivo, human Trop2 cDNA was transfected into the TNBC MDA-MB-231 cell line, which resulted in an approximately four-times increase in Trop2 expression (from about 30,000 to 120,000) [55]. The outcomes showed that the transfected MDA-MB-231 cell line did not change its sensitivity to irinotecan, but significantly enhanced the therapeutic activity of SG.

Another crucial consideration is to minimize collateral damage to normal tissue. Given that Trop2 is expressed in many normal tissues, and Trop2 expression levels in cynomolgus monkeys are similar to those in humans, Cardillo et al. effectively conducted safety studies in cynomolgus monkeys. In the highest dose SG group, monkeys experienced typical SN-38/irinotecan toxicity, severe diarrhea and neutropenia [50]. Another non-clinical trial verified that anti-Trop2 antibodies delivered SN-38 more effectively than irinotecan. SN-38 levels in tumor tissues revealed that SG was 20–136 times higher than irinotecan. Additionally, they noted significantly reduced concentrations of glucuronidated SN-38 (SN-38G) in the serum of animals given SG. SN-38G is a detoxified derivative of SN-38. This suggests that SG can reduce serious adverse events (AEs) relative to irinotecan [51].

#### Datopotamab deruxtecan (Dato-DXd, DS-1062)

Daiichi Sankyo developed Dato-Dxd for the treatment of metastatic breast cancer and metastatic non-small cell lung cancer (NSCLC). The linker of Dato-DXd is highly stable in circulation because it can only release DXd after being cleaved by lysosomal enzymes, which partially solves the issue of early drug release. The longer half-life of Dato-Dxd makes a once-every-three-weeks dosing schedule possible. Moreover, Dato-Dxd has a higher therapeutic index, releasing only 5% of its payload after 21 days, while SG released 90% of its payload after 3 days [52].

In preclinical studies, Dato-Dxd significantly inhibited the proliferation of high Trop2 cell lines but had no inhibitory effect on the proliferation of low Trop2 cell lines. These findings were validated in a Trop2-positive xenograft mouse model. A single dose of 10 mg/kg Dato-Dxd significantly inhibited tumor growth with an inhibition rate of 96%. In contrast, neither datopotamab nor control ADC inhibited tumor growth at the same dose. In mouse and primate models, the safety profile of Dato-Dxd was evaluated. Dato-Dxd caused only minor intestinal or hematopoietic toxicity in rats and monkeys, with no serious changes even at the maximum feasible dose, possibly due to the reduction of off-target toxicity by the stable link employed by Dato-Dxd. However, at doses ≥ 30 mg/kg, pulmonary toxicity was observed in monkey models with cell infiltration, edema, and fibrosis. Hyperpigmentation in the epidermis (not reversible) and corneal lesions were also observed in monkey skin and corneas [56].

Cytotoxic mechanisms of Dato-Dxd on cancer cells include DXd release and bystander effect. The activity of DXd is about 10 times that of SN-38, although DXd may be more effective in interfering with tumor cell DNA replication and recombination, it also carries a higher risk of cytotoxicity. Due to off-target effects, DS-1062 may lead to interstitial lung disease (ILD) in normal tissues [56]. The payload of SG, SN-38, is metabolized and cleared through UGT1A1, and mutations in UGT1A1 to UGT1A1*28 can lead to the accumulation of SN-38 and toxicity [52]. Besides, Trop2 is expressed in some normal epithelial tissues including skin and esophagus, thus rational drug design is essential to minimize the potential risk of targeted toxicity in normal tissues expressing Trop2.

#### SKB-264

SKB-264 has a humanized IgG1 monoclonal antibody (mab), a topoisomerase I inhibitor KL610023 (derived from belotecan), and a cleavable linker. The release of the payload after SKB-264 internalization is proportional to the expression of Trop2 [57]. KL610023 is a new camptothecin analog, which has shown stronger anti-tumor activity in preclinical studies compared to other camptothecin derivatives including belotecan [58]. The linker of SKB264 is modified based on CL2A, and its structure is highly similar to CL2A. The DAR of SKB264 is 7.4, which is similar to SG (DAR of 7.6). However, the conjugation method of SKB264 involves a nucleophilic aromatic substitution reaction between methanesulfonic acid and thiol, unlike other linkers containing thiols that react with albumin’s thiol group causing linker-payload detachment, making SKB264 more stable and reducing side effects. Preclinical studies have shown that the half-life of SKB264 in xenograft mouse models is approximately 57 h, while that of SG in the same model is 14 h, indicating that SKB264 is more stable in vivo compared to SG [59, 60].

#### ESG-401

ESG-401 consists of a topoisomerase I inhibitor SN-38 connected to the humanized anti-Trop2 IgG1 mab via a cleavable linker. The DAR of ESG-401 is 8 [61]. In preclinical studies, ESG-401 has demonstrated excellent safety profile with no observed off-target or off-tumor toxicity in non-human primate studies at high doses and repeated administrations. Additionally, ESG-401 has shown significant anti-tumor activity in a range of Trop2-positive tumor models, with low effective doses and prolonged inhibitory effects.

#### JS-108

JS-108 is intended to treat small cell lung cancer, pancreatic cancer, Trop2 positive TNBC, as well as other solid cancers. The recombinant humanized mab against Trop2 is attached to the tubulysin B analog Tub196 with potential anticancer action via a 2,3-disubstituted long side-chain hydrolysis-resistant linker. JS-108 is now being tested for patients with advanced solid tumors in a phase 1, open-label clinical research to determine its safety, tolerability, PK profile, and effectiveness (NCT04601285) [62].

#### FDA018

Shanghai Fudan-Zhangjiang Bio-Pharmaceutical Co Ltd. produced FDA018, an ADC that targets Trop2 and has an undisclosed payload and linker. FDA018 is now participating in a phase 1 clinical study (NCT05174637) to investigate its safety and pharmacokinetics in advanced solid tumors.

### Others

In addition to ADCs, several other Trop2-targeted therapies are being developed and tested. Trop-2 can interact with multiple proteins such as IGF-1, Claudin-1, and Claudin-7, and has numerous downstream signaling pathways that converge into a complex network. Therefore, small molecule targeted drugs against a single target or monoclonal antibodies blocking a single signaling pathway are not sufficient to inhibit the action of Trop-2. AR47A6.4.2 is a functional Trop2-targeting mab generated by ARIUS’s FunctionFIRST platform. In vitro, AR47A6.4.2 demonstrated cytotoxicity in human cancer cell lines, while in vivo, it displayed anti-tumor activity in human pancreatic cancer models by downregulating the MAPK signal transduction pathway [63]. TrMab-6 is another novel highly sensitive mab against Trop2. TrMab6 strongly induced complement-dependent cytotoxicity (CDC) and antibody dependent cellular cytotoxicity (ADCC) activities against Trop2-overexpression CHO-K1 (CHO/Trop2) and breast cancer cell lines (MCF7, MDAMB231, and MDAMB468) [64]. In parental CHO-K1 and MCF7/Trop2 knockout cells, this activity was not observed. In vivo, experiments on CHO/Trop2 and MCF7 xenografts showed that TrMab-6 significantly reduced tumor growth but had no antitumor activity against parental CHO-K1 and MCF7/Trop2-knockout xenografts. These findings suggest that TrMab-6 has the potential to treat Trop2-expressing breast cancer [64]. In the future, it is recommended to develop various modalities, such as ADCs or chimeric antigen receptor (CAR)-T of TrMab-6, to enhance the effectiveness of antitumor treatment for breast cancer.

Besides, Liu et al. investigated a novel bispecific antibody, F7AK3, that recognizes both Trop2 and CD3. It is a new class of immunotherapy drugs that inhibits tumor growth by recruiting T cells in tumor tissue and activating them. They investigated F7AK3-mediated T cell activation and cytotoxicity in both TNBC cell lines and primary cells in a xenograft TNBC model. They found that cytotoxic efficiency correlated with the level of Trop2 expression in breast cancer cell lines. In conclusion, F7AK3 does not bind to Trop2-negative breast cancer cell lines, nor does it cause any T cell toxicity in Trop2-negative cell lines [65]. Whereas, it remains unanswered whether the Trop2 expression level is related to clinical response and would F7AK3 be appropriate in patients with low Trop2 expression.

Moreover, a nanoparticle that binds to an anti-Trop2 antibody is another component in the future development of Trop2-targeted treatments. In Trop2-expressing TNBC cell lines, the researchers confirmed that doxorubicin-loaded anti-Trop2 antibody conjugated nanoparticles were more toxic than doxorubicin-loaded control nanoparticles without the anti-Trop2 antibody [66]. Others including chimeric Trop2 virus-like particles [67, 68] and anti-Trop2-based photothermal therapy [69] and so on, all of these novel agents are on development, which warrants further clinical evaluation in cancer patients.

## Clinical trials of Trop2-targeted therapy

### Sacituzumab govitecan

To date, SG has been studied in the treatment of several solid tumors, including breast cancer, lung cancer and urothelial cancer. In this chapter, we will provide an overview of the clinical trials of Trop2-targeted therapy in TNBC, HR + breast cancer and other solid tumors (Tables 1, 2 and 3).

**Table 1:** Completed clinical trials involving anti-Trop2 ADCs in breast cancer as monotherapy

Clinical Trials	Phase	anti-Trop2 ADC	Study Design	Endpoints
IMMU-132-01, NCT01631552	I/II	SG	SG in advanced epithelial cancer	TNBC cohort: ORR 33.3%, mDOR 7.7 months, mPFS 5.5 months, OS 13.0 months; HR+/HER2- BC cohort: ORR 31.5%, mDOR 8.7 months, mPFS 5.5 months, mOS 12.0 months
ASCENT, NCT02574455	III	SG	SG vs. TPC in TNBC	mPFS 5.6 vs. 1.7 months; mOS 12.1 vs. 6.7 months; ORR 35.0% vs. 5.0%
EVER-132-001, NCT04454437	II	SG	SG in TNBC	ORR 38.8%, CBR 43.8%, mPFS 5.55 months
NeoSTAR, NCT04230109	II	SG	SG as neoadjuvant treatment in TNBC	pCR rate (using SG alone) 30%
TROPiCS-02, NCT03901339	III	SG	SG vs. TPC in HR+/HER2- BC	ORR 57% vs.38%; PFS 5.5 vs.4.0 months; mOS 14.4 vs.11.2 months
SASCIA, NCT04595565	III	SG	SG vs. TPC in HER2- BC with residual disease after neoadjuvant chemotherapy	Primary: iDFS; Secondary: OS, dDFS, iBCFS
TROPHY-U-01, NCT03547973	II	SG	SG in UC	ORR 27%, mPFS 5.4 months, OS 10.9 months
TROPiCS-03, NCT03964727	II	SG	SG in NSCLC	ORR 17%, mPFS 5.2 months, mOS 9.5 months
TROPION-PanTUMOR01, NCT03401385	I	Daptopotamab Deruxtecan	DS-1062 in NSCLC, TNBC and HR+/HER2- BC	NSCLC cohort: ORR[4 mg/kg,24%(12/50);6 mg/kg,26% (13/50);8 mg/kg,24%(19/80)] TNBC cohort: DOT 2.8 months, ORR 39% (15 PR), DCR 84%(32/38); HR+/HER2-BC cohort: ongoing
A264, NCT04152499	I/II	SKB-264	SKB-264 in solid tumors	NSCLC cohort: mDOT 5.7 months, ORR 44%, mDOR 9.3 months
NCT04892342	I/II	ESG-401	ESG-401 in solid tumors	TNBC cohort: ORR 36%, DCR 64%; HR+/HER2-BC cohort: ORR 62%, DCR 77%

Abbreviations: median (m); overall survival (OS); overall response rate (ORR); progression-free survival (PFS); duration of response (DOR); duration of therapy (DOT); disease control rate (DCR); dose-limiting toxicities (DLTs); recurrence-free survival (RFS); invasive disease free survival (iDFS); distant disease-free survival (dDFS); invasive breast cancer-free survival (iBCFS); time to progression (TTP); time to deterioration (TTD); time to response (TTR); recommended phase II dose (RP2D); maximum tolerated dose (MDT); time to peak (Tmax); half-life time (t1/2); peak plasma concentration (Cmax)

**Table 2:** Ongoing clinical trials involving anti-Trop2 ADCs in breast cancer as monotherapy

Clinical Trials	Phase	anti-Trop2 ADC	Study Design	Endpoints
NCT04647916	II	SG	SG in patients with HER2- BC and brain metastases	Primary: ORR; Secondary: OS, PFS, AEs
NCT04639986	III	SG	SG vs. TPC in HR+/HER2- mBC	Primary: PFS; Secondary: OS, ORR, DOR, AEs
ASCENT-J02, NCT05101096	I/II	SG	SG in japanese participants with advanced solid tumors or TNBC	Primary: RP2D, AEs (phase I); ORR (phase II)
ASCENT-03, NCT05382299	III	SG	SG vs. TPC in TNBC	Primary: PFS, Secondary: OS, ORR, DOR, TTR, AEs
ISIdE, NCT05552001	III	SG	SG in TNBC	Primary: ORR, Secondary: PFS, DOR, CBR, OS
ASCENT-07, NCT05840211	III	SG	SG vs. TPC in HR+/HER2- mBC	Primary: PFS; Secondary: ORR, OS, DOR, AEs
TROPION-Breast01, NCT05104866	III	Datopotamab deruxtecan	DS-1062 VS. TPC in HR+/HER2- BC	Primary: PFS, OS; Secondary: ORR, DOR, DCR
TROPION-Breast02, NCT05374512	III	Datopotamab deruxtecan	DS-1062 vs. TPC in TNBC	Primary: PFS, OS; Secondary: ORR, DOR, TTD
TUXEDO-2, NCT05866432	II	Datopotamab deruxtecan	DS-1062 in TNBC with newly diagnosed or progressing brain metastases	Primary: intracranial response rate; Secondary: PFS, OS
TROPION-PanTumor02, NCT05460273	I/II	Datopotamab deruxtecan	DS-1062 in TNBC and other solid tumors	Primary: ORR; Secondary: DOR, DCR, PFS, OS, TTR, AEs
NCT05347134	III	SKB-264	SKB-264 vs. TPC in TNBC	Primary: PFS; Secondary: ORR, DOR, DCR, OS
NCT05174637	I	FDA018	FDA018 in TNBC and other solid tumors	Primary: DLT, MDT; Secondary: ORR, PFS, DOR, OS, Tmax, t1/2, Cmax

**Table 3:** Ongoing and completed clinical trials involving anti-Trop2 ADCs in breast cancer and other solid tumors as combination therapy

Clinical Trials	Phase	anti-Trop2 ADC	Study Design	Endpoints
ASCENT-04, NCT05382286	III	SG	SG + Pembrolizumab vs. TPC + Pembrolizumab in TNBC	Primary: PFS; Secondary: OS, ORR, DOR
ASCENT-05, NCT05633654	III	SG	SG + Pembrolizumab vs. TPC in TNBC patients who have residual invasive disease after surgery and neoadjuvant therapy	Primary: iDFS; Secondary: ORR, dDFS, RFS, AEs
Saci-IO, NCT04468061	II	SG	SG + Pembrolizumab in PD-L1-negative TNBC	Primary: PFS; Secondary: OS, ORR, DOR
NCT04448886	II	SG	SG + Pembrolizumab in HR+ /HER2 - mBC	Primary: PFS; Secondary: OS, ORR, CBR, TTP, DOR
InCITe, NCT03971409	II	SG	SG + Avelumab in TNBC	Primary: ORR; Secondary: PFS, OS, CBR
Morpheus-panBC, NCT03424005	I/II	SG	SG + Atezolizumab in BC	Primary: ORR, AEs; Secondary: PFS, OS, DCR, DOR
ASPRIA, NCT04434040	II	SG	SG + Atezolizumab in TNBC	Primary: rate of undetectable circulating tumor cfDNA- 6 cycles
ELEVATE TNBC, NCT04958785	II	SG	SG + Magrolimab in TNBC	Primary: DLTs, AEs, PFS, ORR
TROPION-Lung02, NCT04526691	I	Datopotamab deruxtecan	DS-1062 + Pembrolizumab (+ Platinum chemotherapy) in NSCLC	ORR 60% DS-1062 + Pembrolizumab vs.55% DS-1062 + Pembrolizumab + Platinum chemotherapy
BEGONIA, NCT03742102	I/II	Datopotamab deruxtecan	DS-1062 + Durvalumab (+ Paclitaxel) in mTNBC	ORR 66.7% DS-1062 + Duvizumab vs.58.3% Duvizumab + Paclitaxel
TROPION-Breast03, NCT05629585	III	Datopotamab deruxtecan	DS-1062 (+ Durvalumab) vs. TPC in TNBC	Primary: iDFS, Secondary: dDFS, OS, TTD
NCT05445908	II	SKB-264	SKB-264 (+ KL-A167) in TNBC	Primary: AEs, ORR; Secondary: PFS, DOR, DCR
NCT04039230	I/II	SG	SG + Talazoparib in mBC	Primary: DLTs; Secondary: DOR, PFS, OS, TTR
SEASTAR, NCT03992131	I/II	SG	SG + Rucaparib in TNBC and other solid tumors	Primary: DLTs, AEs (phase 1); ORR (phase 2)
NCT05113966	II	SG	SG + Trilaciclib in TNBC	Primary: PFS; Secondary: OS, ORR, CBR
ASSET, NCT05143229	I	SG	SG + Alpelisib in BC	Primary: RP2D; Secondary: pharmacokinetics, ORR

#### Sacituzumab govitecan in triple negative breast cancer

Since SG shows potent anti-tumor activity in preclinical experiments, more efforts are focused on clinical validation. The phase I trial included four patients with metastatic TNBC (mTNBC), one of whom had a 30% reduction in tumor size. The data showed improved response rates and clinical efficacy in TNBC patients at a starting dose of 10 mg/kg. One patient at this dose level experienced grade 3 diarrhea, and the only dose limited toxicity (DLT) was neutropenia [48, 52]. On April 22, 2020, the FDA accelerated the approval of SG for the treatment of patients with mTNBC, based on the data from the IMMU-132-01 trial. IMMU-132-01 was a phase I/II study, investigating 108 mTNBC patients who had received at least two therapeutic regimens. Results showed the median duration of response (mDOR) was 7.7 months (95% CI, 4.9–10.8) and the overall response rate (ORR) was 33.3% (95% CI, 24.6–43.1). Additionally, the OS was 13.0 months (95% CI, 11.2–13.7) and the median progression-free survival (mPFS) was 5.5 months (95% CI, 4.1–6.3). Nausea (67%), diarrhea (62%), fatigue (55%), myelosuppression (74%), and neutropenic fever (9.3%) were the most common AEs. Of the patients, 42.4% and 5.3% had a grade ≥ 3 neutropenia and febrile neutropenia [70, 71]. Interestingly, Homozygous patients with the UGT1A1*28 allele were found to have a heightened susceptibility to neutropenia when administered SG, as indicated by a higher incidence of all-grade neutropenia compared to heterozygous or wild-type individuals. On the other hand, the occurrence of diarrhea was not observed to be elevated in homozygous patients with UGT1A1*28, aligning with prior findings suggesting that allele expression did not significantly impact the prevalence of diarrhea [52]. This study has limitations due to its single-arm nature. Moreover, all cohorts did not have independent response assessment, and a validated Trop-2 immunohistochemistry assay was not accessible. Nevertheless, the encouraging results supported the approval of SG in third-line treatment of mTNBC by FDA.

In light of this signal, the phase III ASCENT trial (NCT02574455) attempted to compared the efficacy of SG to the treatment of physician’s choice (TPC; eribulin, capecitabine, gemcitabine, or vinorelbine) in patients with relapsed or refractory mTNBC who had previously been exposed to paclitaxel. A total of 468 patients without brain metastases were randomly assigned to receive SG or TPC. Patients who received SG and TPC had a mPFS of 5.6 months (95% CI, 4.3–6.3) and 1.7 months (95% CI, 1.5–2.6), respectively (hazard ratio (HR) for disease progression or death, 0.41; *p* < 0.001). The mOS was 12.1 months (95% CI, 10.7–14.0) and 6.7 months (95% CI, 5.8–7.7), respectively (HR for death, 0.48; *p* < 0.001). From these data, we noticed that SG group has a more favorable survival outcome than TPC group. Besides, the ORR was 35% (CR 4%, PR 31%) and 5% (CR 1%, PR 4%), respectively. Neutropenia was the most common AE at grade 3 or higher, followed by leukopenia (10% and 5%), diarrhea (10% and < 1%), anemia (8% and 5%), and febrile neutropenia (6% and 2%), which is consistent with IMMU-132-01 trial [72]. Encouragingly, in patients experiencing a reduction of ≥ 3 grades in neutrophils or ≥ 2 grades in diarrhea, the clinical prognosis is consistent with the total population and is not affected by AEs. In the exploratory analyses, results demonstrated that patients with high, medium, and low Trop2 expression who received SG achieved higher ORRs (44%, 38%, and 22%, respectively) than those who received TPC (1%, 11%, and 6%) [73]. It suggested SG efficacy was independent of Trop2 expression. Moreover, the widespread expression of Trop-2 in mTNBC patients allows for the application of SG without complex test of Trop-2 expression level. This simplifies the treatment process and provides a more convenient treatment option for mTNBC patients. Accordingly, the FDA approved SG for patients with unresectable locally advanced or metastatic TNBC who have previously received ≥ 2 therapeutic regimens. Additionally, ASCENT (NCT02574455) included patients with brain metastases. However, these patients were eliminated from the main analysis population due to subgroup analyses indicating no benefit. Nevertheless, there is a separate study of SG in brain metastases (NCT04647916, Table 2). Subsequently, EVER-132-001 (NCT04454437) is the first clinical trial to investigate the efficacy and safety of SG in Chinese patients with mTNBC, which is a multi-center, single-arm, phase II study. By the cutoff date of August 6, 2021, 80 female patients received ≥ 1 SG dose with a median of 8 treatment cycles. ORR and clinical benefit rate (CBR) were 38.8% (95% CI, 28.06-50.30%) and 43.8% (95% CI, 32.68-55.30%), respectively. The mPFS was 5.55 months (95% CI, 4.14-N/A). Grade ≥ 3 adverse events (AEs) were recorded by 71.3% of patients, with neutrophil count decreased (62.5%) being the most frequent, followed by blood cell count decreased (48.8%) and anemia (21.3%) [74]. The above studies involve in European Caucasian and Asian populations, with consistent conclusions on efficacy and safety, and common and manageable adverse events in clinical practice. More importantly, patients can benefit from SG regardless of Trop-2 expression levels.

The role of SG in the second-line treatment of advanced TNBC has been well established, and is increasingly being considered for (neo)adjuvant treatment as well. NeoSTAR trial (NCT04230109), the other phase II study, is the first to assess SG as a neoadjuvant therapy for patients with local TNBC. The evaluation of lymph node and breast pathologic complete response (pCR) rates were the primary objective. The study included patients with no prior treatment localized TNBC (tumor size ≥ 1 cm, or any size if node positive). A total of 50 patients were enrolled, after four cycles, patients with biopsy-confirmed residual disease, who are considered to have no pCR as the primary endpoint, had the option to receive additional neoadjuvant therapy determined by the treating physician. Most patients (98%, *n* = 49) had completed 4 cycles of SG treatment. The pCR rate in the SG group was 30% (*n* = 15/50, 95% CI, 18-45%). Among 24 patients who received additional neoadjuvant treatment, 6 had a pCR. Of the patients with a germline BRCA mutation (*n* = 8), 7 had surgery directly after SG and 6 had a pCR (86%, 95% CI, 42–99%). The 2-year EFS rate of all patients at a median follow-up of 18.9 months was 95%. The 2-year EFS rate for patients achieving pCR after 4 cycles of SG therapy was as high as 100%, while the 2-year EFS rate for patients with residual lesions after SG therapy was 92%. The most common AEs were nausea (82%, *n* = 41), fatigue (78%, *n* = 39), alopecia (76%, *n* = 38), and neutropenia (58%, *n* = 29). None of the patients discontinued SG therapy due to disease progression or AEs. As of January 8, 2022, no patient experienced disease recurrence. (Table 1) [75]. However, the first interim analysis of Keynote-522 trial, a phase III study of pembrolizumab combined with chemotherapy in neoadjuvant therapy for stage II-III TNBC patients, demonstrated a pCR of 64.8% (95%CI, 59.9–69.5%) [76]. Obviously, the results of NeoSTAR were not as ideal as Keynote-522 trial. This may be because the NeoSTAR study had a smaller sample size and included part of stage I TNBC patients. With addition of PD-1 mab in Keynote-522, the ASCENT-05 study would explore the efficacy of SG combined with pembrolizumab in neoadjuvant and post-surgery residual disease TNBC patients.

#### Sacituzumab govitecan in hormone receptor positive breast cancer

HR+/HER2- is the most common subtype of breast cancer, accounting for approximately 70% of all breast cancer patients. CDK4/6 inhibitors combined with endocrine therapy is the first-line standard treatment for advanced HR+/HER2- breast cancer patients without visceral crisis, but the optimal treatment regimen after progression remains unclear. In the HR+/HER2- cohort of IMMU-132-01, 54 female patientswere enrolled. At a median follow-up of 11.5 months, the mDOR was 8.7 months (95% CI, 3.7–12.7) and the mPFS was 5.5 months (95% CI, 3.6–7.6). The mOS was 12 months (95% CI, 9.0-18.2) and the ORR was 31.5% (95% CI, 19.5-45.6%). Grade ≥ 3 AEs included neutropenia (50.0%), anemia (11.1%), and diarrhea (7.4%). Meanwhile, no treatment-related deaths occurred [77]. Therefore, in patients with previously treated HR+/HER2- breast cancer, SG exhibits potent anti-tumor activity and has a predictable and tolerable safety profile.

After that, a randomized phase III trial (TROPiCS-02) has been completed. In the TROPiCS-02 study, SG was also tested in endocrine resistant HR+/HER2-patients with malignancies that had progressed on a median of three prior chemotherapies. SG would be compared with the treatment of the physician’s choice (TPC). Patients were randomly assigned to receive either SG (*n* = 272) or TPC (*n* = 271) in this trial. When compared to TPC, SG showed an improvement in ORR (57% vs. 38%) and PFS (5.5 vs. 4.0 months; HR 0.66; *p* = 0.0003). Statistics of OS with a median follow-up of 12.5 months disclosed mOS was prolonged by 3.2 months in SG group (14.4 vs. 11.2 months; HR 0.79, *p* = 0.020). Besides, the key grade ≥ 3 AEs (SG vs. TPC) were neutropenia (51% vs. 38%) and diarrhea (9% vs. 1%) [78–81]. According to post hoc analysis, SG can improve the prognosis of HR+/HER2- patients regardless of Trop-2 expression levels, providing a new treatment option for more HR + HER2- metastatic breast cancer patients. Based on this promising result, the FDA approved SG for HR+/HER2- breast cancer patients who have experienced at least two lines of system treatments or endocrine therapy. Whereas, variations in prior treatments and physician-selected chemotherapy in the study may have impacted effectiveness.

Another phase III clinical study, SASCIA (NCT04595565), randomized patients with HER2- breast cancer with residual disease after neoadjuvant chemotherapy (NACT) to SG or treatment of physician’s choice (TPC), is also ongoing. At present, SG was observed a higher AE rate than TPC, including only observations. More dose delays were caused by AEs, particularly the G3-4 AEs rate, which were in line with previous safety profile of SG. The most common AE of grade 3 or above was neutropenia (42.2% in SG and 0% in TPC), followed by leukopenia (28.9% and 0%), nausea (4.4% and 0%), and diarrhea (4.4% and 0%). Using the suggested supportive measures, AEs brought on by SG treatment were effectively managed. The study is continuing as intended (Table 1) [82]. Besides, ASCENT-07 (NCT05840211) is underway in ER + breast cancer, in which SG will be compared to first line chemotherapy (Table 2).

#### Sacituzumab govitecan in other solid tumors

The IMMU-132-01 trial (NCT01631552) we mentioned earlier has confirmed SG in NSCLC (*n* = 47), SCLC (small cell lung cancer, *n* = 50), HR+/HER2 − metastatic breast cancer (*n* = 54), mTNBC (*n* = 108) and mUC (metastatic urothelial cancer, *n* = 45) with anti-tumor activity [70]. To further understand the role of SG in mUC, TROPHY-U-01 study was conducted in patients with relapsed or refractory mUC. The OS and mPFS were 10.9 months (95% CI, 9.0-13.8 months) and 5.4 months (95% CI, 3.5–7.2 months), respectively. The ORR was 27% (31 of 113; 95% CI, 19.5–36.6). Besides, the most common grade ≥ 3 AE was neutropenia (35%), with 6% of patients discontinuing their therapies due to AEs [83]. Based on the TROPHY-U-01 trial (NCT03547973), FDA accelerates approval of SG for the treatment of advanced or metastatic urothelial cancer.

Beyond these, another phase II trial TROPiCS-03(NCT03964727) enrolled 54 patients with metastatic NSCLC. Results showed that in the intention to treat population (*n* = 54), the ORR was 17% and the median onset of responses was 3.8 months. The mOS was 9.5 months (95% CI, 5.9–16.7 months) and the mPFS was 5.2 months (95% CI, 3.2–7.1 months). In terms of safety, as previously reported, grade ≥ 3 AEs included neutropenia (28%), leukopenia (9%), pneumonia (9%), diarrhea (7%), nausea (7%), and fatigue (6%). The conclusion is that SG is well tolerated in many previously treated patients with metastatic NSCLC [84]. However, the results of the EVOKE-01 clinical trial provided us with new insights and considerations. The EVOKE-01 study is a global, multicenter, open-label phase III clinical trial (*N* = 603) that evaluated the efficacy and safety of SG compared to docetaxel in patients with metastatic or advanced NSCLC who had progressed during or after platinum-based chemotherapy and checkpoint inhibitor therapy [85]. The results showed that, compared to the docetaxel group, patients in SG group had a slightly longer OS, but it did not reach statistical significance (mOS 11.1 vs. 9.8 months, HR 0.84). It is worth noting that in patients who do not respond to PD-(L)1 regimens, the mOS in the SG group was 11.8 months, while it was 8.3 months in the docetaxel group (HR, 0.75; 95% CI, 0.58–0.97), indicating a meaningful improvement in OS. However, in patients who respond to PD-(L)1 regimens, there was no significant difference in OS between the two groups [85]. Collectively, the application of SG in NSCLC requires further investigation (Table 1).

### Datopotamab deruxtecan (Dato-DXd, DS-1062)

Followed by SG, several clinical trials have evaluated the efficacy and safety of Dato-Dxd in various solid tumors, including NSCLC, TNBC and HR+/HER2- breast cancer. In the phase I research TROPION-PanTUMOR01 (NCT03401385), patients with advanced solid tumors are being investigated after receiving standard treatment to determine the safety and effectiveness of Dato-Dxd [86]. Dato-Dxd was administered intravenously at 6 mg /kg every 3 weeks in patients with advanced/metastatic TNBC and HR+/HER2- breast cancer. Among them, all patients have received ≥ 2 previous treatment lines. Interim data revealed the ORR of HR+/HER2- breast cancer cohort and TNBC cohort were 26.8% (95% CI, 14.2 ∼ 42.9) and 31.8% (95% CI, 18.6 ∼ 47.6) respectively; while the ORR of topo I-naive subgroup in TNBC cohort was 40.0% (95% CI, 22.7 to 59.4). The mPFS of HR+/HER2- breast cancer cohort and TNBC cohort were 8.3 months (95% CI, 5.5–11.1 months) and 4.4 months (95% CI, 3.0 -7.3 months) respectively; with the median PFS of topo I-naive TNBC subgroup being 7.3 months (95% CI, 3.0–18.0 months).The mOS has not been reached for HR+/HER2- breast cancer cohort, while it was 13.5 months (95% CI, 10.1–16.3 months) for TNBC cohort; and 14.3 months for topo I-naive TNBC subgroup. Of note, 14 patients previously treated with topoisomerase I inhibitor ADC were included in the TNBC cohort, and the observed efficacy in these patients was consistent with the overall TNBC cohort, suggesting that sequential ADC therapy with Dato-Dxd may also achieve certain clinical benefit. The phase III TROPION-Breast01 study of Dato-DXd has also achieved positive results in the treatment of HR+/HER2- breast cancer. The data showed that patients in the Dato-Dxd group had significantly improved PFS (6.9 vs. 4.9 months; HR = 0.63; 95%CI, 0.52–0.76; *p* < 0.0001), and OS also showed a trend towards benefit (HR = 0.84) [87]. This study further supports Dato-Dxd as a new treatment option for advanced HR+/HER2- breast cancer.

In the subgroup of TROPION-PanTUMOR01 study, a total of 180 patients with NSCLC had received DS-1062 at 4 mg/kg (*n* = 50), 6 mg/kg (*n* = 50), and 8 mg/kg (*n* = 80). In the preliminary analysis of the TROPION-PanTUMOR01 trial, Dato-Dxd showed actively anti-tumor effectiveness in patients with advanced/metastatic NSCLC, mPFS and mOS were 6.9 months (95% CI, 2.7–8.8 months) and 11.4 months (95% CI, 7.1–20.6 months), respectively. The most frequent AEs among patients at different doses were nausea (52%) and stomatitis (48%). However, neutropenia (6%) and diarrhea (16%) were rare in Dato-Dxd [88]. Regarding optimal dosage, Dato-Dxd demonstrated anti-tumor activity and safety both in NSCLC and TNBC, with a dosage of 4/ 6/ 8 mg/kg. In addition to being higher in effectiveness and having lower discontinuation rates for AEs, the 6 mg/kg dose was more tolerable than the 8 mg/kg dose. Aside from TROPION-PanTUMOR01 study, TROPION-Lung01 is a global, randomized, multicenter phase III trial evaluating the efficacy and safety of Dato-Dxd (6.0 mg/kg) compared to docetaxel in patients with locally advanced or metastatic NSCLC who have received at least one prior therapy and have no genetic mutations. The dual primary endpoints are PFS and OS. According to the data presented at European Society for Medical Oncology (ESMO) 2023, Dato-Dxd reduced the risk of disease progression or death by 25% compared to docetaxel, with a median PFS of 4.4 months vs. 3.7 months. And the ORR was 26.4% vs. 12.8%. In non-squamous NSCLC patients, the risk of disease progression or death was reduced by 37%, with a mPFS of 5.6 months vs. 3.7 months [89]. This will facilitate the approval of Dato-Dxd for the treatment of locally advanced or metastatic non-squamous NSCLC in adult patients previously treated with systemic therapy. However, it cannot be ignored the dose reduction rate due to any grade AEs is 20% in the Dato-DXd group and 29% in the chemotherapy group, and discontinuing treatment rate is 8% vs. 12%. In later-line therapy, the acceptance of treatment discontinuation and mortality rate requires further discussion.

### SKB-264

SKB264 demonstrates good efficacy and manageable safety in patients with mTNBC and NSCLC previously treated with multi-line therapies. A phase I/II study (NCT04152499) in relapsed or refractory locally advanced/metastatic NSCLC and other tumor types of patients treated with SKB-264 now has interim clinical data. Until February 9, 2023, a total of 43 patients (63% male, median age 58 years) were included. The mDOT was 5.7 months (range 0.5–14.1 months) and the median follow-up was 11.5 months (95%CI, 10.4–12.2 months). Among 39 response-evaluable patients, the ORR was 44% and the mDOR was 9.3 months (range 1.3 to 11.2 months). 67.4% (29/43) of patients experienced grade ≥ 3 AEs. Reduced neutrophil count (32.6%) was the most frequent grade ≥ 3 AE. Moreover, grade 4 AEs occurred only with neutropenia and leukopenia. There were no reports of neuropathy or drug-related ILD, and no discontinuation of treatment or death from AEs. Overall, SKB264 has shown a positive anti-tumor effect in patients with relapsed or refractory locally advanced / metastatic NSCLC, as well as a manageable safety profile. Moreover, SKB-264 has already been the subject of phase III clinical trials in advanced/metastatic NSCLC patients (Table 1) [90]. The updated data on the efficacy and safety of SKB264 in a phase II expansion study for locally advanced or mTNBC were presented at the 2022 SABCS conference. 89.8% of the enrolled 59 patients have received 3 or more prior treatment regimens. The results showed an improved ORR of 53.1%, mDoR of 11.1 months, mPFS of 5.8 months, and ongoing mOS in TROP2 high-expressing patients, with 12- and 24-month OS rates of 65.3% and 57.3%, respectively. The safety profile indicated a 59.3% incidence of grade 3 or higher AEs, with no cases of ILD or grade 3 or higher diarrhea observed, and no treatment related deaths [91]. In comparison, SG treatment for TNBC had an ORR of 33.3% and mPFS of 4.8 months, while Dato-DXd treatment for TNBC had an ORR of 32% and mPFS of 4.4 months. SKB264 achieved the best PFS data for TNBC, with a similar safety profile without ILD events.

### ESG-401

In patients with metastatic or locally advanced solid malignancies, a cohort expansion and dose-escalation phase I/II research (NCT04892332) examined the pharmacokinetics, safety, and anticancer effectiveness of ESG-401. As of February 3, 2023, 35 patients received more than one dose of ESG-401 during escalation at doses of 2–20 mg/kg once Q3W (Regimen A), or 12–16 mg/kg D1,8,15 in a 4-week cycle (Regimen B). Thirty-five patients with refractory/relapsed locally advanced/metastatic solid tumors had, as of February 3, 2023, received less than one dose of ESG-401 during escalation at doses of 2–20 mg/kg once every three weeks (Regimen A) or 12–16 mg/kg every day for four weeks (Regimen B). The most common AE was leukopenia (80%) and the most common grade ≥ 3 AE was neutropenia (31%). No drug-related ILD was reported. Of the 33 patients whose efficacy could be evaluated, 12 were found to have partial responses (PR), and 4 achieved stable diseases (SD) ≥ 24 weeks. The first dose level found PR (16 mg/kg Q3W) and afterward was taken as therapeutically relevant doses (TRD). The ORR and DCR were 36% (4/11) and 64% (7/11) in 11 TRD patients with TNBC, respectively, while 62% (8/13) and 77%/ (10/13) in 13 TRD patients with HR+/HER2-breast cancer. As of the deadline, 11 patients (31%) were still receiving treatment. Three patients were treated for more than 12 months, with the longest treatment duration being 12.9 months (Table 1) [61].

### Combination therapy with Trop2-targeted ADCs

#### Immunotherapy

##### SG + immunotherapy

The novel bispecific CAR-T targeting both TROP-2 and PD-L1 can release high levels of gamma interferon and interleukin-2, exhibiting good anti-tumor activity in gastric cancer xenograft models, which could further enhance the therapeutic efficacy of CAR-T in solid tumors [92]. It suggests TROP2-targeting mab and immune checkpoint may have a synergistic anti-tumor effect in solid tumors. Therefore, SG in combination with pembrolizumab is currently being evaluated in a randomized phase III ASCENT-04 (NCT05382286) trial, compared to treatment of physician’s choice (TPC) combined with pembrolizumab. The primary outcome is PFS. The phase III ASCENT-05 (NCT05633654) trial is conducted in TNBC patients who have residual invasive disease after surgery and neoadjuvant therapy. Similarly, SG combined with pembrolizumab is compared with TPC in this trial. Further two trials are testing the combination of SG and pembrolizumab in PD-L1-negative TNBC (Saci-IO, NCT04468061) and HR+ / HER2-mBC (NCT04448886) patients, both with PFS as the primary endpoint. Besides, patients with mTNBC are being enrolled in the phase II InCITe trial (NCT03971409), which is testing the effectiveness of SG and avelumab combined [93]. Phase I/II (Morpheus-panBC, NCT03424005) and phase II (ASPRIA, NCT04434040) clinical trials are now being conducted with atezolizumab in combination with SG. Additionally, a phase II trial (ELEVATE TNBC, NCT04958785) combining SG with magrolimab, an anti-CD47 IgG antibody, in mTNBC patients is ongoing (Table 3). These data haven’t been published yet, and we are eagerly waiting for promising results.

##### Dato-Dxd + immunotherapy

The potential synergistic activity between ADC and immune checkpoint inhibitors is currently being actively investigated in multiple clinical studies. TROPION-Lung02 (NCT04526691) is a phase I dose-escalation and -expansion study evaluating the safety and tolerability of Dato-Dxd + pembrolizumab ± platinum chemotherapy in patients with NSCLC. As of October 31, 2022, 120 patients had been treated. PD-L1 expression was < 1%, 1-49%, and ≥ 50% in 40%, 33%, and 26% of patients, respectively. The most common AEs were stomatitis (45%) and nausea (45%). Grade ≥ 3 AEs were developed in 61% of patients, the most common of which were neutrophil count decreased (8%) and amylase increased (8%). Twelve patients developed drug-related ILD. AEs that were serious, associated with discontinuation, or associated with death occurred in 31%, 24% (16% associated with Dato-Dxd), and 6% of patients, respectively. The ORR for Dato-Dxd + pembrolizumab duplex and Dato-Dxd + pembrolizumab + platinum chemotherapy triad were 60% (95% CI, 36-81%) and 55% (95% CI, 39-70%), respectively. Overall, a combination of Dato-Dxd with pembrolizumab with/without platinum showed a tolerable safety profile and also demonstrated satisfactory anti-tumor activity in NSCLC [94].

Thereafter, two clinical trials were to evaluate Dato-Dxd in combination with immunotherapy in TNBC patients. The BEGONIA trial (NCT03742102) examined Dato-Dxd in combination with durvalumab with or without paclitaxel as the first-line treatment for mTNBC patients. The study showed that the ORR of duvizumab combined with Dato-Dxd was improved (66.7%) compared to duvizumab plus paclitaxel (58.3%). Most patients (73%) had PD-L1 low tumors (5%), and duvizumab combined with Dato-Dxd achieved therapeutic efficacy regardless of PD-L1 status [95]. Another phase III clinical trial, TROPION-Breast03 (NCT05629585), was conducted to explore the efficacy of Dato-Dxd ± durvalumab versus TPC in TNBC patients without pCR following neoadjuvant therapy (Table 3).

##### SKB-264 + immunotherapy

In patients with unresectable locally advanced, metastatic, or recurrent TNBC who have not previously received systemic therapy, SKB-264 with or without KL-A167 (a PD-L1 inhibitor) is being tested in the phase II clinical trial known as NCT05445908 to determine its safety, tolerability, and anti-tumor efficacy. The primary endpoints are AEs and ORR (Table 3).

#### Targeted therapy

##### SG + PARP inhibitor

Given that both SG and PARP inhibitors prevent DNA replication and repair, there may be a synergistic effect between the two medications. The payload of SG is a topoisomerase I inhibitor, and the PARP inhibitor blocks the topoisomerase I cleavage complex induced by topoisomerase I inhibitors, which unfortunately results in dose-limited myelosuppression when these therapies are used in combination. A phase I/II clinical trial (NCT04039230) evaluated SG in combination with talazoparib in patients with mTNBC [96]. The trial has entered phase II and the primary goal is to evaluate DLTs. In addition, there is another phase I/II trial (SEASTAR, NCT03992131) studying the combination of SG and Rucaparib in patients with solid tumors including mTNBC, and the primary endpoint is also DLT (Table 3) [97].

##### SG + CDK4/6 inhibitor

Trilaciclib is a selective and reversible CDK4/6 inhibitor that is administered intravenously prior to chemotherapy and actively preserves hematopoietic stem and progenitor cells, as well as immune system function (myeloprotection) from chemotherapy-induced damage. In February 2021, trilaciclib was approved by the FDA for use in patients with extensive small cell lung cancer before receiving treatment with a platinum-containing/etoposide or topotecan regimen to reduce the incidence of chemotherapy-induced myelosuppression. According to preliminary data from the phase II clinical trial (NCT05113966), in patients with unresectable locally advanced or metastatic TNBC, administration of trilaciclib prior to SG treatment meaningfully reduced AEs associated with ADCs (Table 3) [98].

##### SG + PI3K inhibitor

The ASSET trial (NCT05143229) is assessing the efficacy of the combination of SG and the PI3K inhibitor alpelisib in patients with metastatic or locally recurrent HER2-negative breast cancer. Since 8–25% of TNBC patients have PI3K activating mutations, the researchers hypothesized that the combination of the two drugs would have a synergistic effect [99, 100]. The recommended phase II dose (RP2D) of the combination is the primary outcome (Table 3).

## Challenges and directions

Although Trop2-targeted ADCs have shown promising therapeutic effects on a variety of solid tumors, there are still many challenges that need to be addressed. Firstly, the safety management of Trop2-targeted ADCs cannot be generalized due to affection by various factors, such as the physiological functions of non-tumor tissue targets, the nature of linkers, the types and quantities of cytotoxic agents, as well as immunogenicity, off-target toxicity, bystander effects, etc. Among them, SG developed adverse reactions such as neutropenia and gastrointestinal toxicity, while SKB264 mainly has hematological toxicity and oral mucositis, and Dato-DXd requires attention to special AEs such as oral mucositis, ocular toxicity, and infusion-related events [41, 101]. In addition, due to the expression of Trop-2 in tissues such as the skin, upper digestive tract mucosa, and ocular surface conjunctival epithelium, ocular toxicity and oral mucositis are non-tumor targeted toxicities that need to be closely monitored during the application. Thus, active prevention and promptly prophylaxis are particularly important. For example, for patients with neutropenia, granulocyte colony-stimulating factor (G-CSF) may be considered as secondary prophylaxis. For patients with evidence of decreased UGT1A1 enzyme activity, such as early onset of acute or unusually severe adverse reactions, close monitoring should be conducted, and treatment may need to be temporarily or permanently discontinued. For gastrointestinal adverse reactions, prophylactic use of antiemetics or antidiarrheal agents may be initiated before treatment.

Secondly, despite the promising efficacy of Trop2-targeted therapy, many patients still develop primary and secondary drug resistance, and the underlying mechanisms are not well understood. Recently, Leshchiner I et al. disclosed the potential resistance mechanisms of SG. They suggested the loss of TROP2 may lead to inability to recognize cancer cells and drives primary resistance. The TROP2 T256R missense mutation results in decreased binding ability of TROP2 with antibodies and the TOP1 E418K missense mutation causes the loss of the active site for SN-38 [102]. Besides, the defection in endocytic pathway may hinder the entry of ADCs into cells and impaired lysosomal function or decreased lysosomal proteolytic activity can result in ineffective degradation of ADCs [103]. These details need further explorations to provide precise guidance for patient selection and next-generation ADC development. The strategies for combating drug resistance include optimizing structural design, developing new ADCs such as bispecific antibodies, combination with targeted therapy or immunotherapy, and utilizing predictive biomarkers to estimate efficacy. It is rational to combine DNA damage and repair Inhibitors with ADC that have different mechanisms of action and minimal overlapping toxicities. Additionally, the combination of ADC with immunotherapy can enhance anti-tumor immune responses and improve clinical outcomes. Dual-target ADC drugs can overcome drug resistance issues caused by decreased expression of a single target. In exploratory analysis of ASCENT and TROPiCS-02 trial, the efficacy of SG was independent of TROP2 expression. Meanwhile, homologous repair defect was founded to be associated with efficacy of TROP2 ADCs in preclinical research, which requires validation in clinic practice.

## Conclusions

Trop2 has been identified as a major regulator of multiple processes involved in the development of tumors. Although the receptor is found in all breast cancer subtypes, Trop2 is highest expressed in TNBC. Moreover, Trop2 is a prospective target for cancer therapy since it is over-expressed in the majority of malignancies while being down-expressed in healthy tissues. Following the FDA’s approval of SG for TNBC and HR+/HER2-breast cancer, other Trop2-targeted ADCs such Dato-Dxd and SKB-264 are also coming to the forefront. To break the limitations of tumor heterogeneity on Trop2-targeted therapy, clinical trials of anti-Trop2 ADCs in combination with immunotherapy, targeted therapy and chemotherapy regiments are underway. To assess the effectiveness of Trop2-targeted therapy in patients with early-stage illness, the current trial is also being undertaken in neoadjuvant and adjuvant therapy. There are also studies to improve the effectiveness and safety of ADCs by replacing them with new payloads and linkers. In general, Trop2 is a novel therapeutic target for breast cancer patients who have not responded to conventional chemotherapy. To expand the more significant therapeutic benefits of Trop2-targeted therapy beyond breast cancer to other Trop2-positive malignancies, sustained efforts are needed.

## Data Availability

No datasets were generated or analysed during the current study.

## Abbreviations

Trop2: Human trophoblastic cell surface antigen 2
TNBC: Triple negative breast cancer
HR: Hormone receptor
HER2: Human epidermal growth factor receptor 2
TACSTD2: Tumor-associated calcium signal transducer 2
TX: Transmembrane helix
ICD: Intracellular domain
ECD: Extracellular domain
PIP2: Phosphatidyl-inositol 4,5-bisphosphate
PH: Pleckstrin-homology
RIP: Regulation of intramembrane proteolysis
ADAM: A disintegrin and metalloprotease
OS: Overall survival
DAR: Drug-antibody ratio
TI: Therapeutic index
DXd: DNA topoisomerase I inhibitor exatecan derivative
CDC: Complement-dependent cytotoxicity
DCC: Antibody dependent cellular cytotoxicity
FS: Event-free survival
SMO: European Society of Medical Oncology
ACT: Neoadjuvant chemotherapy
OT: Duration of therapy
CR: Disease control rate
R: Partial response
SD: Stable disease
TRD: Therapeutically relevant dose
